# Anatomical reconstruction of the patellar tendon using the fascia lata attached to the iliac bone following resection for soft tissue sarcoma: A case report

**DOI:** 10.3109/03009734.2012.689379

**Published:** 2012-10-30

**Authors:** Hiroatsu Nakashima, Masahiro Yoshida, Kentaro Miyamoto

**Affiliations:** Department of Orthopedic Surgery, Aichi Hospital, Aichi Cancer Center, Okazaki, Japan

**Keywords:** Fascia lata, patellar tendon, reconstruction, soft tissue sarcoma

## Abstract

A new reconstruction of the patellar tendon was performed in a 43-year-old patient who lost tendon and tibial tuberosity after a wide tumor resection for low-grade myofibroblastic sarcoma of the parapatellar tendon. In this technique, the patellar tendon was anatomically reconstructed using a fascia lata attached to the iliac bone. The iliac bone was fixed to the tibial bony trough with absorbable screws, and the fascia lata was fashioned into three branches: the central branch was folded through the tunnel in the patella, and the medial and lateral branches were tagged to the medial and lateral retinaculum, respectively, around the patella. The skin defect was covered by the bilateral head of the gastrocnemius flap and a split-thickness skin graft. At the 3-year follow-up, the active range of motion of the knee joint was 0 to 110 degrees. The functional result according to the Musculoskeletal Tumor Society scoring system was 97%. Radiographs showed that the grafted bone was united well to the tibial bone, and the grafted fascia was confirmed as a dark band on MRI. There was no evidence of disease and no complaint of the donor site. This procedure allows for the reconstruction of the patellar tendon in the original location. To our knowledge, this reconstructive procedure of the patellar tendon using the fascia lata attached to the iliac bone has never been reported in English literature.

## Introduction

Low-grade myofibroblastic sarcoma represents a distinct atypical myofibroblastic tumor, often with fibromatosis-like features. It occurs predominantly in adult patients with a slight male dominance. The tumor shows a wide anatomic distribution; however, the extremities and the head and neck region appear to be preferred locations. Clinically, local recurrences are common, whereas metastases only occur rarely (1). Therefore, wide surgical resection of the tumor is important.

We report a case of low-grade myofibroblastic sarcoma, involving the region adjacent to the tibial tuberosity and the patellar tendon, in a 43-year-old woman. The defect of the patellar tendon following limb-sparing wide tumor resection was reconstructed by the fascia lata attached to the iliac bone and covered with the gastrocnemius flap. To our knowledge, this type of reconstructive procedure has never been reported in English literature.

## Case report

A 43-year-old woman visited a general clinic with a painless tumor of the right anterior knee region and underwent excision of the tumor under local anesthesia. The pathological diagnosis was spindle cell sarcoma, and the lesion recurred locally. She was referred to our hospital 2 months after the initial excision.

**Figure 1.**
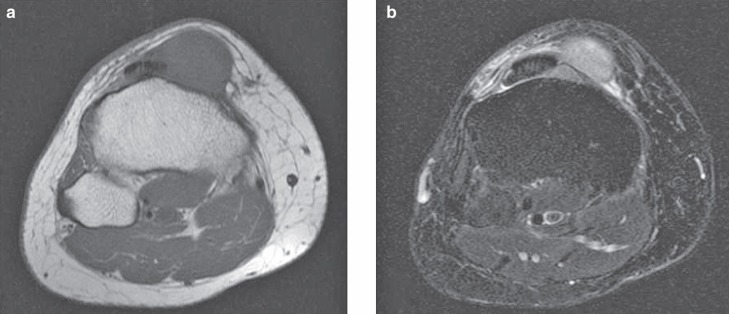
Magnetic resonance images of the tumor at the initial presentation. The tumor showed low signal intensity on T1-weighted images (a) and high signal intensity on fat-suppressed T2-weighted images (b). The tumor was located closely to the patellar tendon, and a part of the tumor crept beneath the tendon. A high signal intensity area, suggesting edema, was seen around the tumor.

**Figure 2.**
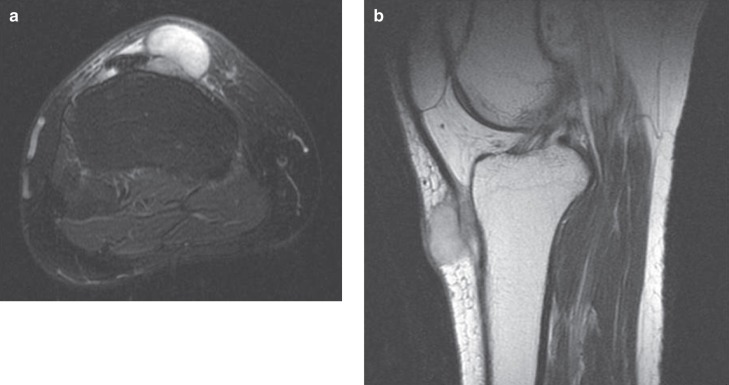
MRI after preoperative radiation therapy. The size of the tumor was reduced, and the high signal intensity area was decreased.

Physical examination of the right anterior knee region revealed the transverse excision scar and tumor at the medial side of the tibial tuberosity. The tumor was elastic and soft, and movable, and its longitudinal diameter was 3 cm. Diffuse swelling was seen around the tumor. The range of motion of the knee joint was normal. Plain radiographs showed no abnormality. Magnetic resonance imaging (MRI) showed a well-margined mass adjacent to the patellar tendon and that a part of the tumor crept under the patellar tendon. The tumor showed low signal intensity on T1-weighted images and high signal intensity on T2-weighted images. Fat suppression T2-weighted images showed a high signal intensity area around the tumor, suggesting edema (Figure 1). The swelling of the surrounding tissue was severe. Thus, if additional wide resection with the safety surgical margin including this edema area were to be established, the extent of skin and subcutaneous tissue excision was expected to be considerable. Therefore, preoperative radiation (total dose 25 Gy) was performed in order to reduce the surgical margin. MRI after preoperative radiotherapy showed the reduction of the tumor size (reduction rate 34%), and the extent of high signal intensity around the tumor was reduced on fat suppression T2-weighted images (Figure 2). We established a 3 cm skin margin from the edema area, and performed wide resection of the tissues surrounding the tumor, including almost the full length of the patellar tendon (the patellar tendon was resected transversely 1 cm from the inferior pole of the patella), the infrapatellar fat pad, and the tibial tuberosity.

### Patellar tendon reconstruction

**Figure 3.**
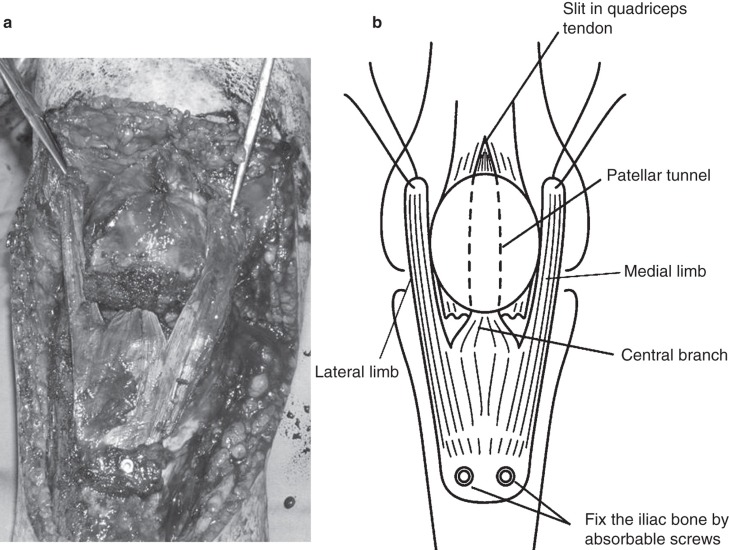
Reconstruction of the patellar tendon using the fascia lata attached to the iliac bone: intraoperative photo (a) and schematic drawing (b). The central branch of the graft was passed through an 8–9 mm longitudinal tunnel and then through a slit in the quadriceps tendon.

A 4 × 10 cm portion of the fascia lata attached to iliac bone was harvested. The surgical technique adopted was similar to that described in *Campbell's operative orthopaedics* (2). The bone trough in the tibia was made with an oscillating saw about 4 cm distal to the joint line. Contouring of the corticocancellous iliac bone was performed to fit the tibial bony trough, and the bony portion was fixed with two 4.5 mm absorbable cortical screws. The fascia lata portion was fashioned into three branches, with the central third consisting of half of the width. This central branch was 8–9 mm in diameter. A Kirschner wire was passed through the central part of the patella to make a tunnel. An 8–9 mm reamer was passed over the Kirschner wire. A whipstitch was made with a non-absorbable suture in the central branch, and this central branch was passed through the tunnel, exiting through a slit in the quadriceps tendon. Multiple interrupted non-absorbable sutures were placed through the graft in the soft tissue of the inferior pole of patella and at the edges of the quadriceps tendon (Figure 3). The appropriate graft length and tension were determined as follows. The position of the inferior pole of the patella was situated at the upper portion of the intercondylar notch at 45 degrees knee flexion. A lateral view radiograph of the knee joint was obtained to confirm the height of the patella compared with the opposite side. Patellar tracking was checked carefully. The medial and lateral branches of the graft to the medial and lateral retinaculum were tagged, respectively, using non-absorbable sutures. The skin defect was covered by the bilateral head of the gastrocnemius flap and a split-thickness skin graft. The pathological diagnosis of low-grade myofibroblastic sarcoma was made.

### Postoperative rehabilitation

**Figure 4.**
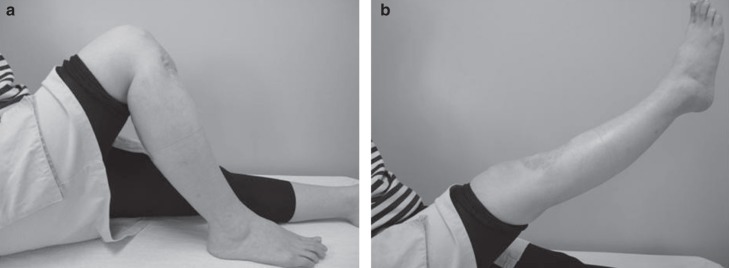
Clinical photo taken 3 years after the operation showed active knee flexion of 110 degrees (a). The patient is able to raise her leg with an extension lag of 5 degrees (b).

**Figure 5.**
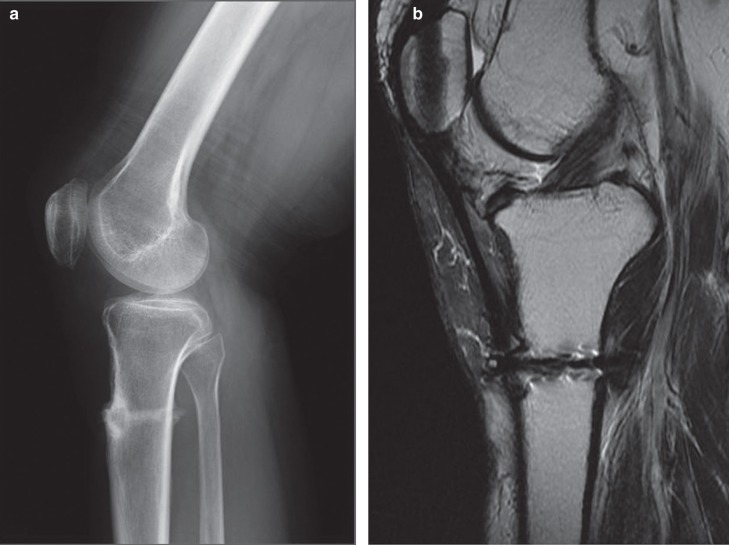
Lateral-view radiograph (a) and MRI (b) taken 3 years after the operation. Bone union is obtained well and the Insall–Salvati index is 1.6 (the opposite knee is 1.1). Therefore, the patella alta is seen. The graft is shown as a dark band on the T2-weighted image (b).

Postoperative swelling in the lower leg was severe, and peroneal nerve palsy developed, but improved 3 months after operation. The patient was initially treated with a splint for 3 weeks with the knee in full extension. Continuous passive motion was initiated to move the knee between 0 and 30 degrees, and this range was gradually increased. At the same time, isometric quadriceps strengthening exercise was begun. After 4 weeks postoperatively, weight-bearing to tolerance with crutches was allowed until sufficient motion and strength allowed for unassisted ambulation. Physical examination at 3 years postoperatively showed active knee motion of 0 to 110 degrees. She was able to raise her leg with an extension lag of 5 degrees (Figure 4). The functional result according to the Musculoskeletal Tumor Society (MSTS) scoring system (3) was 97%; pain, function, emotional acceptance, supports, and walking were 5 points, respectively, and gait was 4 points. Radiographs showed that the grafted bone was united well to the tibial bone, and the grafted fascia was confirmed as a dark band on T1- and T2-weighted images (Figure 5). There was no evidence of systemic or local recurrence and the donor site was clinically unaffected.

## Discussion

There have been very few reports on reconstruction of the patellar tendon after a wide tumor resection such as in this case (4,5). Fukui et al. (4) reported a case with soft tissue sarcoma close to the patellar tendon. The patellar tendon and tibial tuberosity were resected due to wide resection of the tumor. The patellar tendon was reconstructed with a graft composed of the ipsilateral hamstring tendon and iliotibial tract. Both ends of the graft were fixed in the bone tunnels in the patella and tibia by screw fixation. Twenty months postoperatively, full range of the knee joint was achieved without extension lag. Machens et al. (5) reported a case with prepatellar myxofibrosarcoma. Limb-saving radical tumor resection was achieved by resection of the caudal quadriceps muscle, patella, collateral ligaments, and patellar tendon. The large defect was covered by a free myocutaneous latissimus dorsi flap. The patellar tendon was replaced by insertion of the flap, and the muscular flap origin was connected to the remaining quadriceps muscle. The patient was able to extend his knee joint actively without any external support 3 months after operation, but clinical photographs, obtained 9 months later, showed the range of his knee to be about 70 degrees flexion and with an extension lag of about 10 degrees. Peyser and Makley (6) reported a new reconstruction of the patellar tendon in a 10-year-old boy with synovial sarcoma in the prepatellar tendon region. The inferior pole of the patella, patellar tendon, and tibial tuberosity were resected completely along with the patellar tendon. The biceps tendon attached to the fibular head was harvested, and the fibular head was fixed to the trough made from resection of the tibial tuberosity using a screw. The biceps tendon was sutured proximally to the quadriceps tendon and reinforced with the semitendinosus tendon and reversed quadriceps tendon. In this technique the reconstructed patellar tendon was located in the exact anatomical location of the original tendon, similar to our procedure. Fukui et al. (4) reported that success in reconstruction of the patellar tendon was dependent on the strength and anchoring of the graft, and the anatomic graft location. Noyes et al. (7) reported the biomechanical analysis of a variety of graft tendons used in ligament reconstruction. The patellar tendon–bone graft was the strongest, and its central or medial portions of one-third width had greater strength (159% to 168%) compared with the anterior cruciate ligaments. The semitendinosus and gracilis tendons were stronger (70% and 49%, respectively) compared with the anterior cruciate ligaments, and the iliotibial tract and fascia lata had strength of 44% and 36%, respectively. However, the fascia lata 45 mm in width had strength of 104% of the anterior cruciate ligament, and wider grafts from the fascia lata could increase the strength. In our case, the harvested fascia lata attached to the iliac bone was 4 mm in width, but the fascia was folded back, so the graft fascia appeared to be suitable for the strength required from the biomechanical analysis of Noyes et al (7).

Regarding anchoring of the graft, it is important to obtain firm graft-to-bone healing in reconstruction of the patellar tendon, and it is important to locate the reconstructed graft in the exact anatomical location of the original tendon to regain the normal biomechanics of the patellofemoral joint and to decrease the risk of subsequent degenerative changes in the patella (4,6). We reconstructed the patellar tendon using the fascia lata attached to the iliac bone for anatomical reconstruction. A similar technique using the tendo calcaneus allograft appears in *Campbell's operative orthopaedics* (2). At the 3-year follow-up, our patient was able to raise her leg with an extension lag of 5 degrees and obtained good results with 97% of the MSTS score.

This study is a case report, and a larger series of patients treated using this technique would be needed to evaluate the utility of this method. However, from this experience, the present procedure may be recommended for the treatment of neglected ruptures of the patellar tendon or traumatic defect of the tendon.
